# Effects of Apple Juice Concentrate, Blackcurrant Concentrate and Pectin Levels on Selected Qualities of Apple-Blackcurrant Fruit Leather

**DOI:** 10.3390/foods2030430

**Published:** 2013-09-12

**Authors:** Lemuel M. Diamante, Siwei Li, Qianqian Xu, Janette Busch

**Affiliations:** Department of Wine, Food and Molecular Biosciences, Lincoln University, Lincoln 7647, Canterbury, New Zealand; E-Mails: Siwei.Li@lincolnuni.ac.nz (S.L.); Qianqian.Xu@lincoln.ac.nz (Q.X.); Janette.Busch@lincoln.ac.nz (J.B.)

**Keywords:** response surface methodology, apple juice, blackcurrant, pectin, physicochemical qualities, ascorbic acid, fruit leather

## Abstract

A study was conducted to determine the effects of different levels of apple juice concentrate (AJC), blackcurrant concentrate (BCC) and pectin on the moisture content, water activity, color, texture and ascorbic acid content of apple-blackcurrant fruit leather using the response surface methodology. The results showed the moisture content increased with increasing pectin level and with greater increases at higher AJC and BCC levels while the water activity increased with increasing pectin level and with increasing AJC level, at low pectin levels, but with decreasing AJC, at high pectin levels. The chroma decreased with increasing pectin level and with lower values at the middle AJC level. The puncturing force decreased with increasing AJC level but with a lower value at the middle pectin level. Lastly, the ascorbic acid content increased with increasing BCC level regardless of AJC and pectin levels. There is a need to reduce the drying temperature or time of apple-blackcurrant fruit leather just enough to bring the water activity closer to 0.60, thereby increasing the moisture content resulting in higher product yield.

## 1. Introduction

Apples are one of the most consumed fruits worldwide and are consumed fresh or in processed forms such as jam, juice or dried. Apples contain over 84% water, a variety of vitamins (except vitamin B complex), minerals (K, Mg, Ca, Na), trace elements (Zn, Mn, Cu, Fe, B, F, Se, Mo) and have a high fiber content. Due to the varied and well balanced composition of apples, they have the potential to prevent digestive cancers, colon and liver cancers, coronary heart disease, lung function disorder and asthma [1].

Blackcurrants from New Zealand have a significant presence in the domestic and international market, due to their attractive taste and aroma and nutritional benefits. Blackcurrants are 1 cm in diameter, are very dark purple in color (black), with a glossy skin and are highly nutritious because they are high in vitamin C, polyphenols and anthocyanins, at the same time they are low in calories and sodium; so they have been called the “king of berries” [2]. Consumption of blackcurrants can be a good way to prevent cancer, improve vision, control diabetes, improve circulation, control inflammation, antimicrobial effects and slow the growth of aging effects [3].

Fresh fruit is not easy to store for a long time after harvest so instead of being consumed fresh, drying could be a good way of processing fruit. Drying of fruits is becoming popular due to its simplicity and low cost. Many fruits can be dried whole, such as grapes, berries, apricot, or as slices such as mango, pawpaw, kiwifruit. They can also be peeled, sliced, cored, blended into puree and dried into fruit leathers. Fruit leather is becoming popular in the international market. Fruit leathers are mainly eaten as snacks. They can also be an ingredient in a product [4]. Packaged dried fruits can be stored for several months. Generally, fruit leather is made by the dehydration of fruit puree or mixture of fruit concentrate into a thin, soft and flat layer. It can be dried in an oven or in direct sunlight. Usually, the ingredients used are fruit juice or concentrate, pectin and glucose syrup or sugar. Many types of fruits can be used for making fruit leather such as guava, mango, pear, strawberry, kiwifruit, pineapple [5,6,7,8,9,10]. Apple can be made into fruit leather by using apple juice concentrate (AJC) instead of glucose and sucrose. In this way, the AJC could be used to give a natural sweet taste to the fruit leather. Addition of blackcurrant concentrate (BCC) to the apple fruit leather would enhance the nutritional quality of the product. Moreover, incorporation of pectin would improve the physicochemical and sensory properties of the product [5,7,9,10,11].

Many qualities such as moisture content, water activity, color, texture and ascorbic acid content of fruit leather can be affected by the levels of AJC, BCC and pectin. Both moisture content and water activity are key factors affecting the storage, shelf life and food safety of fruit leather [12,13]. The color of the fruit leather is an important characteristic which can influence the consumer when the purchasing the product. Texture is a critical property of processed food like dried food, which can affect the acceptability of the product. A dried product with significant amount of ascorbic acid would be advantageous.

Response surface methodology (RSM) is one of the most relevant multivariate techniques for analytical optimization. RSM is a collection of statistical and mathematical techniques that have been used successfully in developing, improving and optimizing processes, products, or systems [14]. RSM enables a reduction in the number of experimental trials needed to evaluate the effect of interactions between the variables on the response and to generate large numbers of information, thus, saving time and labor. RSM has been widely used for optimizing processes dehydrated fruit products [15,16,17,18,19]. 

The objective of this study was to determine the effects of the levels of apple juice concentrate, blackcurrant concentrate and pectin on the moisture content, water activity, color, texture and ascorbic acid content of apple-blackcurrant fruit leather. The RSM was used to obtain the quadratic models for describing the effects of the independent variables on the dependent variables. This is a preliminary study and will serve as the basis for further study to produce apple-blackcurrant fruit leather at optimal ingredients levels.

## 2. Experimental Section

### 2.1. Materials

Granny Smith apples and apple pectin powder (high methylester (64% esterification), slow set) were obtained from a local supermarket in Christchurch, New Zealand. Because of the unavailability of fresh apples, old season’s fruits that had been in storage in central cool stores for about six months were used in the study. The apples were stored in a chiller at 4 °C until used in the experiments. The apples were peeled, sliced, cored and blended into puree. Apple juice concentrate (AJC) (69.5°Brix) was obtained from a local supplier while the blackcurrant concentrate (BCC) (63°Brix) was procured from New Zealand Pharmaceuticals, Ltd., Palmerston North, New Zealand.

### 2.2. Preparation of Apple-Blackcurrant Puree Mixture

The apples were peeled, sliced, cored and blended into a puree together with the AJC, BCC and pectin following the formulation shown in Table 1. The blending process was carried out at high speed for 3 min to get a smooth mixture. A total of 400 g puree mixture was made for each run. The puree mixture was poured into an aluminum tray with a non-stick surface and inside dimensions of 30 cm × 20 cm × 1 cm. About 315 g of puree mixture filled up the aluminum tray.

### 2.3. Hot Air Drying Experiments

The hot air dryer used was the same dryer used in Diamante *et al*. (2010) [20]. The trays, which contained the fruit puree mixtures were placed in the middle and upper part of the dryer, and a temperature data logger was placed in the middle of the dryer. The drying time was 16 h, at an average drying temperature of 70 ± 1°C and an air velocity of 0.20 m/s flowing perpendicularly to the sample. The drying conditions used was obtained from preliminary experiments on a puree mixture with AJC, BCC and pectin middle levels formulation to produce fruit leathers that would easily peel off from the drying tray, indicating the correct moisture content. The drying temperature, ambient temperature and relative humidity were monitored using data loggers (Tinytag Ultra2, United Kingdom) for the duration of the experiment.

### 2.4. Moisture Content Determination

At the end of the experiments, the moisture content of the initial fruit puree mixture and dried samples was determined as using an air oven (Watson Victor, Ltd., New Zealand) at 105 °C. Representative pieces of fruit leather were obtained from the samples as well as the fruit puree mixture and dried following a standard method (Method 984.25) [21]. The moisture contents of the different samples were calculated on a percent dry basis and the average values of quadruple samples were used.

### 2.5. Water Activity Measurement

Water activity was measured six times per treatment using fruit leather cut into approximately 2 mm × 2 mm pieces, using an Aqua Lab water activity meter CX-2 (Decagon Devices, Inc., Washington, DC, USA). Results were expressed in mean water activity per treatment at a temperature of 21.9 ± 0.4 °C. A water activity for the fruit leather below 0.60 would indicate microbial stability for the product. A low water activity would give a dry and tough fruit leather.

### 2.6. Color Properties Determination

The color values (CIE L*, a* and b*) of the different fruit leathers were measured with a Minolta Reflectance Chroma Meter CR-210 (Minolta, Japan) as in Diamante *et al.* (2010) [20]. The instrument was calibrated before each measurement with a white ceramic tile (L* = 98.06, a* = −0.23, b* = 1.88). The samples cut into 2 mm × 2 mm pieces to fill a petri dish and then measured eight times at different points. The chroma of the samples were calculated using the equation below,

Chroma = [(a*)^2^ + (b*)^2^]^½^ (1)

where a* and b* are color values of the samples.

A high chroma reading would indicate a deep red purple color for the fruit leather.

### 2.7. Texture Measurements

The textural property of the fruit leather was determined by measuring the force needed to puncture the fruit leather sheet using a texture analyzer (Texture Analyzer Model: TA-XT plus, Serial No: 10 781, Stable Micro System, Surrey, UK) equipped with a 5 kg load cell. A heavy duty platform (HDP/90) with a hole in the center was used to support the fruit leather sheet. A 500 g stainless steel cylinder with a hole in the center was placed on top of the sample to hold it in place. A 2 mm cylindrical probe was used to puncture the sample. Test speed were set to 1.0 mm/s, trigger force to 5 g and travel distance of the probe to 10.0 mm. The cylindrical probe was brought down very close to the sample then the test was started and run until it punctured the sample. The different fruit leathers were measured 12 times at different points. Collected data for puncturing force (N) were analyzed in XTRAD Dimension software from Stable Micro Systems and were expressed as mean values per sample. A low puncturing force indicates a soft fruit leather while a high value would be a tough product.

### 2.8. Ascorbic Acid Measurements

The ascorbic acid contents of the apple pulp, fruit puree mixture and dried samples were measured by titration using a 2,6-dichloroindophenol and following a standard method (Method 967.21) [21]. Measurements were automated using a modified Metrohm titrimetric method (Application bulletin No. 98/2e). The method used a Pt Titrode connected to a 670 Titroprocessor with sample changer with a 16-position 100 mL beaker carousel. Data capture and equipment control was performed using a Tiamo software version 1.2.41 (Metrohm AG, Switzerland). Triplicate measurements were undertaken on each sample.

### 2.9. Experimental Design

A Box-Behnken RSM design was used to derive mathematical models for describing the effects of the independent variables on the dependent variables (Myers *et al.*, 2009, [14]). The three independent variables were AJC level (X_1_), BCC level (X_2_) and pectin level (X_3_). Three levels of each of the three independent variables were chosen for the study and coded as −1, 0 and 1 (Table 1). The dependent variables determined were moisture content (Y_1_), water activity (Y_2_), chroma (Y_3_), puncturing force (Y_4_) and ascorbic acid content (Y_5_).

**Table 1:** Box-Behnken response surface methodology design experiments on apple-blackcurrant fruit leather as affected by apple juice concentrate (AJC), blackcurrant concentrate (BCC) and pectin levels

Coded Factors			Uncoded Factors		
AJC Level	BCC Level	Pectin Level	AJC Level (%)	BCC Level (%)	Pectin Level (%)
−1	−1	0	20	3	2
1	−1	0	40	3	2
−1	1	0	20	9	2
1	1	0	40	9	2
−1	0	−1	20	6	0
1	0	−1	40	6	0
−1	0	1	20	6	4
1	0	1	40	6	4
0	−1	−1	30	3	0
0	1	−1	30	9	0
0	−1	1	30	3	4
0	1	1	30	9	4
0	0	0	30	6	2
0	0	0	30	6	2
0	0	0	30	6	2
0	0	0	30	6	2

Note: −1, low level; 0, middle level; 1, high level.

### 2.10. Data and Statistical Analyses

The data were analyzed using Design Expert 8 (Stat-Ease, Minneapolis, MN, USA) to obtain the quadratic mathematical model, as shown below,

Y = a_0_ + a_1_X_1_ + a_2_X_2_ + a_3_X_3_ + a_4_X_1_X_2_ + a_5_X_1_X_3_ + a_6_X_2_X_3_ +a_7_X_1_^2^ + a_8_X_2_^2^ + a_9_X_3_^2^ (2)

where: Y = dependent variable (quality); a_0_, a_1_, a_2_, a_3_, a_4_, a_5_, a_6_, a_7_, a_8_, a_9_ = coefficients; X_1_ (AJC level), X_2_ (BCC level) and X_3_ (pectin level).

The probability level from the analyses of variance was used as the basis for the statistical significance of the coefficients for the different independent variables.

Using the derived mathematical model for each dependent variable, values were obtained for various levels of AJC, BCC and pectin and then used in making the surface plots using the SigmaPlot 12.0 (Systat Software Inc., San Jose, CA, USA).

## 3. Results and Discussion

### 3.1. Qualities of Apple-Blackcurrant Fruit Leather at Different Conditions

The results of the RSM experiments on the moisture content, water activity, chroma, puncturing force and ascorbic acid content for the apple-blackcurrant fruit leather are shown in Table 2. Generally, the products have higher moisture content with increasing apple juice concentrate (AJC), blackcurrant concentrate (BCC) and pectin levels, higher water activity and lower chroma with increasing pectin level, lower puncturing force with increasing AJC level and higher ascorbic acid content with increasing BCC level. Table 3 shows the coefficients of the quadratic mathematical models for the different qualities of apple-blackcurrant fruit leather at different AJC, BCC and pectin levels. The results show that the linear coefficients of AJC, BCC and pectin levels had a highly significant effect while the 2-factor interaction of the three factors had a significant effect on the moisture content of the apple-blackcurrant fruit leathers. These results showed that the pectin level has the major effect on the moisture content but the AJC and BCC levels have an interacting effect on the pectin level. The water activity of the products was highly significantly affected by the linear coefficient of pectin level and the 2-factor interaction of AJC and pectin levels. The linear coefficient of pectin level had a highly significant effect and the quadratic coefficient of AJC level had a significant effect on the chroma of the fruit leathers. The puncturing force of the products was highly significantly affected by the linear coefficient of AJC level and the quadratic coefficient of pectin level. The linear coefficient of BCC level had a significant effect on the ascorbic acid content of the fruit leathers. These results will be discussed further in the succeeding sections.

**Table 2:** Mean results of the response surface methodology experiments on the qualities of apple-blackcurrant fruit leather as affected by apple juice concentrate (AJC), blackcurrant concentrate (BCC) and pectin levels.

AJC Level	BCC Level	Pectin Level	Moisture Content	Water Activity	Chroma	Puncturing Force	AA Content
(%)	(%)	(%)	(% dry basis)	(no units)	(no units)	(N)	(mg/100 g dry matter)
20	3	2	25.89	0.423	10.72	25.91	21.52
40	3	2	26.11	0.424	7.92	9.28	22.04
20	9	2	26.02	0.408	11.39	29.36	38.98
40	9	2	29.18	0.431	5.26	9.78	27.97
20	6	0	20.79	0.269	16.14	27.62	27.14
40	6	0	21.69	0.358	19.86	13.75	33.08
20	6	4	25.46	0.448	4.16	27.13	30.73
40	6	4	29.23	0.402	7.52	18.29	31.62
30	3	0	21.14	0.313	16.61	22.60	23.74
30	9	0	21.67	0.350	11.18	25.78	24.57
30	3	4	25.06	0.451	3.93	18.23	24.32
30	9	4	28.36	0.477	4.59	19.32	23.72
30	6	2	26.16	0.441	6.02	12.59	27.44
30	6	2	27.42	0.433	6.77	13.15	28.17
30	6	2	27.23	0.433	5.48	11.89	27.31
30	6	2	26.61	0.432	5.47	12.88	27.07

Note: N, Newtons; AA, ascorbic acid.

### 3.2. Effect of AJC, BCC and Pectin Levels on Moisture Content

**Figure 1:**
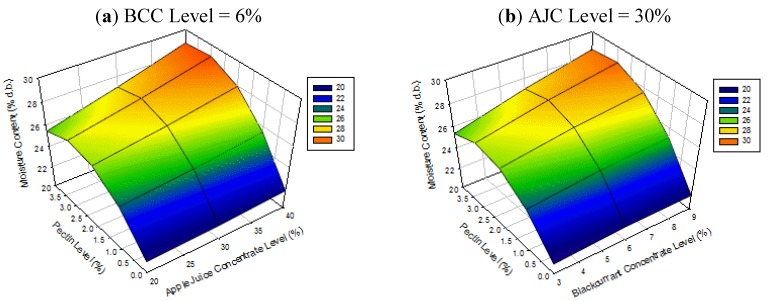
Surface plots for moisture content of apple-blackcurrant fruit leather as affected by apple juice concentrate (AJC) and pectin levels (**a**) and blackcurrant concentrate (BCC) and pectin levels (**b**) with the third factor set at the middle level (legend shows the range of values on the response surface).

Figure 1 shows the surface plots for moisture content of apple-blackcurrant fruit leathers as affected by AJC, BCC and pectin levels. The results suggest that the moisture content of apple-blackcurrant fruit leathers increases with increasing pectin level and with greater increases at higher AJC and BCC levels showing the interaction effects (Table 3). These results were in contrast with those of Phimpharian *et al.* (2011) [10] for the effect of pectin level on moisture content of pineapple leather. The difference might be due to the heating of the pineapple fruit puree mixture, lower pectin level used (0.5% to 1.5%) or the type of fruit used. However, a previous study found that the pectin level (1% to 3%) did not affect the moisture content of mango leather [11]. The use of higher levels of pectin resulted in a product with higher moisture content probably due to the presence of AJC and BCC altering the product water binding property of the product.

**Table 3:** Coefficients of the quadratic mathematical model for moisture content, water activity, chroma, puncturing force and ascorbic acid content of apple-blackcurrant fruit leather using different levels of apple juice concentrate (AJC), blackcurrant concentrate (BCC) and pectin.

Coefficients	Moisture Content	Water Activity	Chroma	Puncturing Force	Ascorbic Acid Content
	(% dry basis)	(no units)	(no units)	(N)	(mg/100 g dry matter)
a_0_	26.85500 ***	0.43475 ***	5.93500 *	12.62750 **	17.49750
a_1_	1.00625 **	0.00838	−0.23125	−7.36500 ***	−0.45750
a_2_	0.87750 **	0.00688	−0.84500	1.02750	2.95250 *
a_3_	2.85250 ***	0.06100 ***	−5.44875 ***	−0.84750	0.23250
a_4_	0.73500 *	0.00550	−0.83250	−0.73750	−2.88250
a_5_	0.71750 *	−0.03375 **	−0.09000	1.25750	−1.26250
a_6_	0.69250 *	−0.00275	1.52250	−0.52250	−0.35750
a_7_	0.09000	−0.02088 *	2.86500 *	3.08500	3.34250
a_8_	−0.14500	0.00763	0.02250	2.87000	−3.21250
a_9_	−2.65250 ***	−0.04463 ***	3.12000 *	5.98500 **	−0.19750

Note: Y = a_0_ + a_1_X_1_ + a_2_X_2_ + a_3_X_3_ + a_4_X_1_X_2_ + a_5_X_1_X_3_ + a_6_X_2_X_3_ + a_7_X_1_^2^ + a_8_X_2_^2^ + a_9_X_3_^2^; Y = quality; X_1_ = AJC level; X_2_ = BCC level; X_3_ = Pectin level; *** Significant at 0.1% level; ** Significant at 1% level; * Significant at 5% level.

### 3.3. Effect of AJC and Pectin Levels on Water Activity

**Figure 2:**
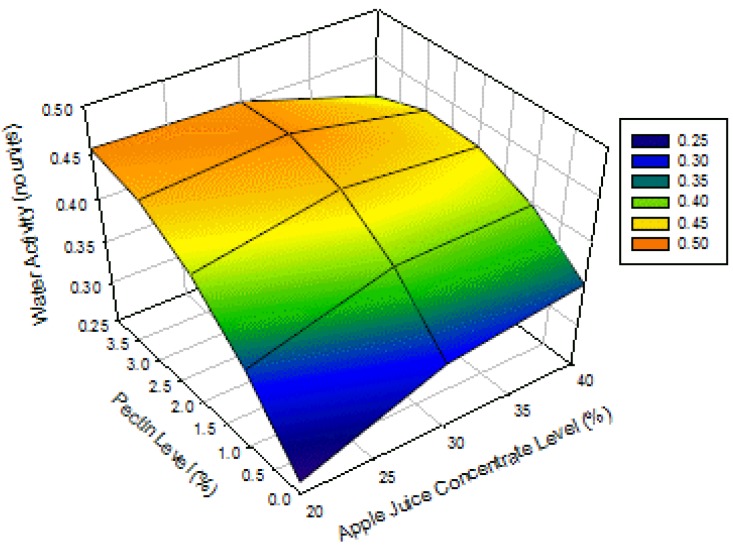
Surface plot for water activity of apple-blackcurrant fruit leather as affected by apple juice concentrate and pectin levels with a blackcurrant concentrate level of 6% (legend shows the range of values on the response surface).

The surface plot for water activity of apple-blackcurrant fruit leathers as affected by AJC and pectin levels is shown in Figure 2. The water activity of the products increases with increasing pectin level and with increasing AJC level, at low pectin levels, but with decreasing AJC at high pectin levels. This phenomenon is due to the quadratic and interaction effects of both AJC and pectin levels (Table 3). These results contrasted those of Phimpharian *et al.* (2011) [10] for the effect of pectin level on water activity of pineapple leather. The difference might be due to the heating of the pineapple fruit puree mixture, the lower pectin level used (0.5% to 1.5%) or the type of fruit used. When there is no pectin in the fruit leather, the water activity of the samples increased with AJC level due to the presence of higher amounts of sugar that thereby bound more water to the food matrix but when the pectin level increased to about 4% the water binding property of the product may have likely changed. There is a need to further study the phenomenon of why the water activity increased with pectin level for this product. This will be done in the next optimization studies. The authors are not in the position at present to fully explain this phenomenon. 

### 3.4. Effect of AJC and Pectin Levels on Chroma

**Figure 3:**
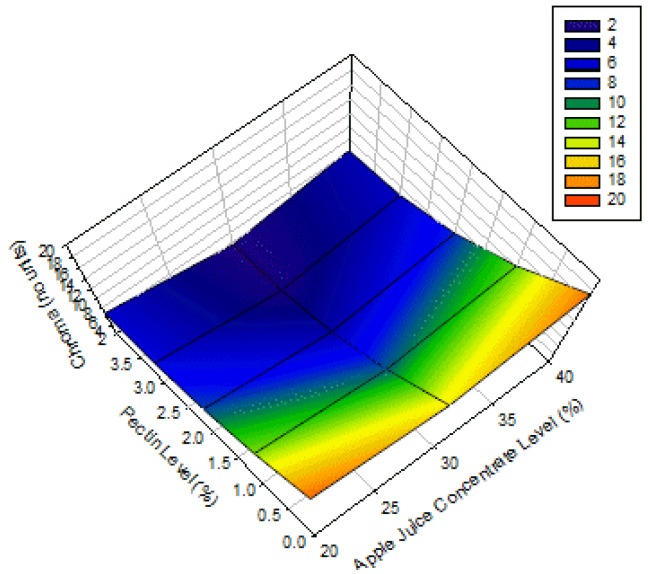
Surface plot for chroma of apple-blackcurrant fruit leather as affected by apple juice concentrate and pectin levels with a blackcurrant concentrate level of 6% (legend shows the range of values on the response surface).

Figure 3 shows the surface plots for the chroma of apple-blackcurrant fruit leathers as affected by AJC and pectin levels. The chroma of the products decreases with increasing pectin level. The middle AJC level gave a lower chroma for the products due to its quadratic effect. These results were in contrast to those of Phimpharian *et al.* (2011) [10] for the effect of pectin level on chroma of pineapple leather. Moreover, this difference might be due to the heating of the pineapple fruit puree mixture, lower pectin level used (0.5% to 1.5%) or the type of fruit used. There is an optimum AJC level in the fruit leather which gave it lower degree of redness. The high levels of pectin probably lessened down the effect of blackcurrant color on the fruit leathers.

### 3.5. Effect of AJC and Pectin Levels on Puncturing Force

**Figure 4:**
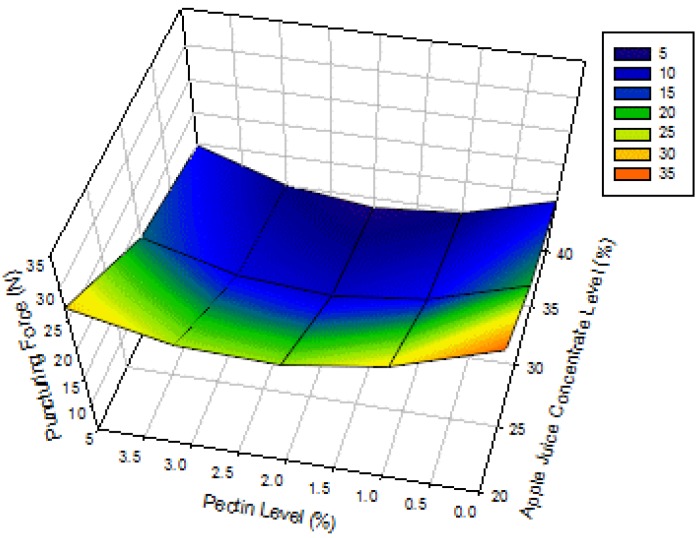
Surface plots for puncturing force of apple-blackcurrant fruit leather as affected by apple juice concentrate and pectin levels with a blackcurrant concentrate level of 6% (legend shows the range of values on the response surface).

The surface plot for the puncturing force of apple-blackcurrant fruit leathers as affected by AJC and pectin levels is shown in Figure 4. The results show that the puncturing force of apple-blackcurrant fruit leather decreases with increasing AJC level but has a lower value at the middle pectin level. The result observed for the pectin level was due to its quadratic effect (Table 3). Huang and Hsieh (2005) [7] also reported that the compressive force/hardness of pear leather decreases with increasing corn syrup level (0% to 8%) regardless of pectin level (16% to 24%). However, Phimpharian *et al.* (2011) [10] found that the tensile force of pineapple leather increased with glucose and pectin levels. The difference might be due to the heating of the pineapple fruit puree mixture, lower pectin level used (0.5% to 1.5%) or the type of fruit used. In addition, the puncturing force (downward force on the fruit leather until it is punctured) is slightly different from the tensile force (stretching force on the fruit leather until it breaks). The higher levels of AJC resulted in fruit leathers with higher amounts of sugar, which probably softened the products. There was an optimum level of pectin that would give softer fruit leathers.

### 3.6. Effect of Blackcurrant Level on Ascorbic Acid Content

**Figure 5:**
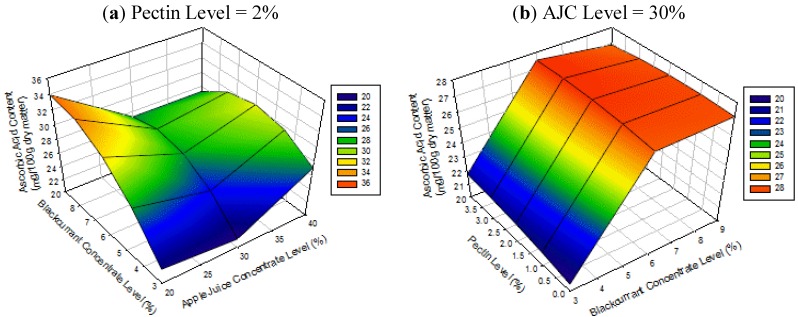
Surface plots for ascorbic acid content of apple-blackcurrant fruit leather as affected by blackcurrant concentrate (BCC) and apple juice concentrate (AJC) levels (**a**) and pectin and BCC levels (**b**) with the third factor set at the middle level (legend shows the range of values on the response surface).

Figure 5 shows the surface plot for the ascorbic acid content of apple-blackcurrant fruit leathers as affected by AJC, BCC and pectin levels. The results show that the ascorbic acid content of the products increases with increasing BCC level regardless of AJC and pectin level. This result was expected since the greater the amount of BCC in the fruit leather the higher is the ascorbic acid content. The initial amount of ascorbic acid in the apple pulp was only 20.59 ± 2.00 mg/100 g dry matter because these apples were in storage for six months as pointed out in the previous section. The increase in the amount of ascorbic acid was not considerable probably due to the low levels of BCC (3% to 9%) and the drying conditions (70 °C for 16 h) used. Diamante and Yamaguchi (2012) [18] reported ascorbic acid contents of 50 to 124 mg/100 g dry matter for dried apple cubes soaked in 10% to 30% BCC level before drying.

### 3.7. Implication of the Results on Apple-Blackcurrant Fruit Leather Processing

Based on the results, the results showed the moisture content increased with increasing pectin level and with greater increases at higher AJC and BCC levels while the water activity increased with increasing pectin level and with increasing AJC level, at low pectin levels, but with decreasing AJC, at high pectin levels. The chroma decreased with increasing pectin level and with lower values at the middle AJC level. The puncturing force decreased with increasing AJC level but was lower value at the middle pectin level. Lastly, the ascorbic acid content increased with increasing BCC level regardless of AJC and pectin levels.

Intermediate moisture foods (IMF) like dried fruits and fruit leathers generally have a moisture content between 11% and 67% (dry basis) and water activity of around 0.60 [22,23]. A water activity of 0.60 is the lowest level at which there is no microbial proliferation [13]. IMF products are foods with a moisture content higher than that of dry foods and are edible without rehydration, and shelf stable without refrigeration during distribution and storage [24]. Dried fruits and fruit leathers generally must have a soft chewy texture and this would be achieved with a higher moisture content but with a water activity that is low enough to inhibit microbial growth. The results show that the apple-blackcurrant fruit leathers produced have microbial stability since their water activity is below 0.60. However, the drying conditions at 70 °C for 16 h need to be modified to reduce either the drying temperature or time just enough to bring the water activity closer to 0.60 thereby increasing the moisture content resulting in higher product yield. It must be noted that the products were within the range of IMF since their moisture content ranged from 21% to 29% dry basis.

## 4. Conclusions

The RSM was successfully applied in understanding the effects of AJC, BCC and pectin levels on the physicochemical and nutritional qualities of apple-blackcurrant fruit leather. The moisture content which ranged from 21.14% to 29.18% dry basis, increased with increasing pectin level and with greater increases at higher AJC and BCC levels due to the effects of their interaction. The water activity ranging from 0.313 to 0.477, increased with increasing pectin level and with increasing AJC level at low pectin levels but with decreased AJC at high pectin levels due to the effects of their interaction. The chroma, which ranged from 3.93 to 19.86, decreased with increasing pectin level and had a lower value at the middle AJC level due to a quadratic effect. The puncturing force ranging from 9.28 to 29.36 N, decreased with increasing AJC level but had a lower value at the middle pectin level due to the interaction effect. The ascorbic acid content, which ranged from 21.52 to 38.98 mg/100 g dry matter, increased with increasing BCC level regardless of AJC and pectin level. There is a need to reduce the drying temperature or time for apple-blackcurrant fruit leather just enough to bring the water activity closer to 0.60, thereby increasing the moisture content and resulting in higher product yield.
